# The Over-Extended Mind? Pink Noise and the Ethics of Interaction-Dominant Systems

**DOI:** 10.1007/s11569-018-0325-x

**Published:** 2018-11-01

**Authors:** Darian Meacham, Miguel Prado Casanova

**Affiliations:** 1 Department of Philosophy, Maastricht University, Grote Gracht 90-92, 6211 Maastricht, SZ Netherlands; 2 BrisSynBio, a BBSRC/EPSRC Synthetic Biology Research Centre, Life Sciences Building, Tyndall Avenue, Bristol, BS8 1TQ UK; 3 UWE, Bristol, Coldharbour Lane, Bristol, BS16 1QY UK; 4 Department of Health and Social Sciences, UWE, Bristol, Coldharbour Lane, Bristol, BS16 1QY UK

**Keywords:** Extended mind thesis, Cognitive artefacts, Pink noise, Machine-human hybrid, Responsible research and innovation, Distributed cognition, Responsibility, Interaction-dominant systems, Enhancement, Human enhancement technology, Value-sensitive design, Noise

## Abstract

There is a growing recognition within cognitive enhancement and neuroethics debates of the need for greater emphasis on cognitive artefacts. This paper aims to contribute to this broadening and expansion of the cognitive-enhancement and neuroethics debates by focusing on a particular form of relation or coupling between humans and cognitive artefacts: interaction-dominance. We argue that interaction-dominance as an emergent property of some human-cognitive artefact relations has important implications for understanding the attribution and distribution of causal and other forms of responsibility as well as agency relating to the actions of human-cognitive artefact couplings. Interaction-dominance is both indicated and constituted by the phenomenon of “pink noise”. Understanding the role of noise in this regard will establish a necessary theoretical groundwork for approaching the ethical and political dimensions of relations between human cognition and digital cognitive artefacts. We argue that pink noise in this context plays a salient role in the practical, ethical, and political evaluation of coupling relations between humans and cognitive artefacts, and subsequently in the responsible innovation of cognitive artefacts and human-artefact interfaces.

Labour appears, rather, merely as a conscious organ, scattered among the individual living workers at numerous points in the mechanical system; subsumed under the total process of the machinery itself, as itself only a link in the system, whose unity exists not in the living workers, but rather in the living active machinery, which confronts his individual, insignificant doings as a mighty organism. ([1], p. 693).

The individual becomes the mere spectator of the results of the functioning of the machines, or the one who is responsible for the organisation of technical ensembles putting the machines to work. ([2], p. 132).

## Introduction

There is growing recognition in the literature on cognitive enhancement and neuroethics of the need for greater attention to the role of cognitive artefacts in the technological intervention into and alteration of cognitive processes. Fasoli [3] has argued for the need for greater consideration of cognitive artefacts in neuroethics and has developed a taxonomy of relationships between cognitive artefacts and cognitive processes [4]. Likewise, Heersmink [5] has argued for the broadening of neuroethics and cognitive enhancement debates to include more consideration of cognitive artefacts, including emerging technologies such as transcranial stimulation and neuro-prosthetics (e.g. [6]) but also greater reflection on “environmental objects and structures”. Heersmink [7] has also developed a multi-dimensional framework for conceptualising integration between cognitive artefacts and human agents. The broadening and greater inclusivity of these descriptive and normative debates to consider a broad range of cognitive artefacts as enhancement technologies have been motivated by developments in what can broadly be referred to as 4E (embodied, embedded, extended, enactive) approaches to cognition (see, for example, [8, 9]). Subsequently, this has furthered the encounter between the debates in the area of cognitive enhancement and those in 4E cognition (see, for example, [10]). In short, if some or all cognitive artefacts are considered forms of enhancement technology, then the form of cognition at issue falls within the domain of 4E approaches. Our aim here is to contribute to this discussion and the encounter between these two fields.

There remains however a lack of sustained engagement both concerning the epistemology of cognitive artefacts in the enhancement debate, and the potential ethical and political challenges arising from the increasing pervasiveness of digital cognitive artefacts [11] in the fields of 4E (or situated) cognition. It seems clear that discussions of enhancement and cognitive enhancement in particular will increasingly centre around hybrid, human-artefact, cognitive systems, and specifically human + digital cognitive artefact systems.^1^ Prospective technologies and scenarios for human cognitive enhancement increasingly implicate hybrids of organic cognitive systems (brains) and digital cognitive artefacts (digital technologies), sometimes called cognitive computing.^2^ The world’s largest edu-business, Pearson [12], one of the world’s largest computing companies, IBM [13], Facebook, Amazon, Google, and Microsoft^3^ have shown strong interest in the development and production of cognitive computing systems applications for use in the educational market, business, government, healthcare, education, and other sectors. On the basis of these interests, there is a common vision of how machine intelligence might perform as cognitive-enhancement technology in various settings.

Consequently, addressing the epistemological as well as ethical and political questions issuing from cognitive artefacts is one of the most important tasks for the debates concerning responsible research and innovation (RRI) in human enhancement technologies. This, more specifically, is how we hope to contribute to the encounter between discussions in cognitive enhancement and those in 4E cognition. RRI is defined by the European Commission as an “approach that anticipates and assesses potential implications and societal expectations with regard to research and innovation, with the aim to foster the design of inclusive and sustainable research and innovation” [14]. It has been adopted as a research and support initiative within large techno-science and innovation funding programmes by many international and national funders including but not limited to the European Commission’s €80 billion Horizon2020 Programme, the UK’s EPSRC (Engineering and Physical Science Research Council),^4^ and the Dutch NWO, where RRI is a flagship programme.^5^ While each funder defines RRI (or sometimes just RI, i.e. responsible innovation) in a slightly different fashion, there are clear overarching commonalities. There is an expanding literature on this approach to bringing societal, ethical and political concerns directly into the research funding and subsequent innovation process(es). These debates, though significant, are outside the scope of this paper, which nonetheless situates itself within the scope of RRI as broadly defined above (and in notes 5 and 6).

## Cognitive Coupling

Relations between humans and digital cognitive artefacts can be characterised as *cognitive coupling* where there is communication or information flow within the coupled system (human-cognitive artefact). The title of this paper, “the over-extended mind”, refers to a phenomenon wherein (1) the cognitive coupling between a human and a cognitive artefact can be described as “interaction-dominant” (a term that we will define below); and (2) the interaction-dominance is ethically and politically significant for how we understand responsibility and agency. The “over-extended mind”, we contend, has consequences pertaining to the responsible innovation and value-sensitive design of those cognitive artefacts that could potentially form intraction-dominant systems with human minds and bodies.

Specifically, we argue that interaction-dominance as an emergent property of some human-cognitive artefact couplings has ramifications for the attribution of agency and responsibility in a fashion that is not discussed in the existing literature. While the characteristic of interaction-dominance can be manifest in relations with all sorts of cognitive artefacts, we think that the ethical and political salience comes of the fore in the discussion of digital cognitive artefacts that make use of algorithms (processes or sets of rules used in calculations or other problem-solving processes).^6^ Thus, there are specific implications for discussions about responsible innovation or value-sensitive design of digital cognitive artefacts and specifically human – digital cognitive artefact interfaces. Consequently, we argue that in view of the potential scenario of the “over-extended mind”, cognitive distance or disruption in the flow of information between certain kinds of cognitive artefact and their human users can be considered a design-virtue and a key element to consider in responsible innovation. To put this another way: building in some forms of noise qua disruption of information flow could be an aspect of value sensitive design in the innovation of human – digital cognitive artefact interfaces. Noise can in some instances be an epistemic virtue. Correspondingly, accessibility, durability, and intensity of information flow, all dimensions of integration between artefact and human agent, can in some cases be undesirables.

Interaction-dominance in human-cognitive artefact couplings or systems is both indicated and constituted by the related phenomenon of “pink noise”: a sub-type of the general concept of noise. Thus, pink noise performs an important heuristic role in identifying and understanding interaction-dominant systems and the over-extended mind phenomenon. To understand the status and role of pink noise, and consequently interaction-dominant systems, we need to first examine how the general concept of noise operates in the loop of interactions which constitutes the flow of information between human and artefacts. This will establish the theoretical groundwork for approaching the ethical and political dimension of relations between human cognition and digital cognitive artefacts. As pink noise is central to the constitution of interaction-dominance and subsequently the over-extended mind, it plays a significant role in the practical, ethical, and political evaluation of coupling relations between humans and cognitive artefacts.

The main body of this paper will thus focus on characterising interaction-dominance in its relation to pink noise in the context of human-cognitive artefact coupling. We conclude with a further discussion of some of the ethical and political dimensions of interaction-dominant systems, already alluded to above. We situate our discussion of the ethical and political dimensions in the context of ongoing discussion concerning cognitive artefacts and distributed morality (e.g. [5, 18]). Thus, one important aspect of what we hope to do here is a kind of translation work from discussions in cognitive science and philosophy of mind to discussions concerning responsible innovation and value-sensitive design.

### The Terms of Engagement

Before proceeding to the analysis of pink noise in its relation to interaction-dominance, it will be helpful to clarify our usage of several key ideas that have already been mentioned above without sufficient elaboration. A *cognitive artefact* is an “artificial device designed to maintain, display or operate upon information” [19] in order to “functionally contribute to the performing of a cognitive task” [20]. The development of 4E approaches to cognition in the philosophy of mind has reinforced the role of cognitive artefacts in cognitive processes. *4E approaches to cognition* emphasise the importance of embodied engagement with the natural, social, and technological milieu as a fundamental aspect of human cognitive processes. There is a rich and growing literature in this field, and while there is a rather wide heterogeneity of approaches, it does not seem unfair to say that there is broad agreement that even our basic cognitive processes are technologically or artefactually mediated, structured, and scaffolded (e.g. [8, 9, 21–23]). What is often at stake in debates under the umbrella of 4E cognition is the extent or degree to which cognitive processes are extended beyond the body (into the environment) and hence whether it is more appropriate to think of cognition as extending into the external environment, or somewhat less dramatically scaffolded by it. Heersmink [5, 11] points out that extended cognition should not be thought about in binary all or nothing terms. Rather, he suggests that it is more appropriate to think of a spectrum of extension relating to the “kind and intensity of information flow between agent and scaffold, the accessibility of the scaffold, the durability of the coupling between agent and scaffold.” As we shall explain, *interaction-dominance* represents an extreme end of this spectrum, where the epistemological, functional, and potentially (though not necessarily) phenomenological delineations between agent and artefact (scaffold) are obscured, precisely due to the dominance of interactions between agent and artefact over discrete agglomerated actions or processes. An interaction-dominant system is one where it is not possible to distinguish discrete causal cognitive components from one another because the organisation of the system arises in the interactions. Consequently, cognition processes pertaining to such couplings should not be analysed as discrete functions of encapsulated molecules, neurons, neural structures, behaviours or other modules without considering their context or mutual interactions.

*Noise* is a phenomenon (or set of related phenomena) present in any conceivable information channel [24], and consequently in every cognitive process. As a working definition, we can say that noise involves irregularities, interference, and distortions in the communication between the target properties in the environment and the sensory signal as well as in perceptual or cognitive processes. Cognitive relations between humans and cognitive artefacts will thus, *de jure*, involve noise. In brief: noise is a given in information channels; information channels are involved in all cognitive processes; thus the extension, regardless of robustness and intensity of cognitive processes into cognitive artefacts or other aspects of the built or natural environment, and subsequently an extension of cognitive processes with the aim of enhancing or augmenting them will involve noise.

Noise in cognitive processes originates from at least two sources. Internally, noise emanates from variability, for instance, noise in neural activation, as when neurons trigger differently on two occasions, despite the same relevant initial conditions. Externally, it arises from inadequate environmental conditions, e.g. listening a conversation close to a busy motorway. In this sense, noise refers to “small (and frequent) fluctuations” [25] which may disturb the achieved stability of a supposed, in this case cognitive, system. A system is considered robust when it resists noise. Arguments in favour of “developmental noise”, “noise-induced order” and “noise-oriented behaviour” maintain that the structural resilience of a system to noise may help individuals or systems gain the ability to adapt to the environment or achieve a higher level of functionality. We think that the role of noise is an under-explored but central dimension to understanding the cognitive, ethical, and eventually social impact of human-artefact coupling. Despite the emerging body of literature concerning digital cognitive artefacts, for example, Heersmink on their taxonomy [20], dimensions of integration [7], and metaphysics [26] and Fasoli’s work on neuroethics [3], there is no account of how noise might affect new models of technology-mediated cognition. In this paper we are particularly interested in the role of pink noise (a sub-type of the umbrella concept noise) as both indicating and constituting interaction-dominant human-artefact cognitive systems. To understand the relation between interaction-dominance and pink noise we need to scrutinise how *noise* operates in the coupling dynamics between human cognizing systems and digital cognitive artefacts that are integrated into and can indeed be said to enhance human cognition.

The idea that noise plays a salient and constitutive role in this way faces some challenges. A first and fundamental challenge issues from the debate within cognitive science concerning whether the appearance of pink noise does indicate an interaction-dominant system. Second, there is the question of whether the epistemological, ethical, and political questions raised by demonstrating that some cognitive coupling may lead to the emergence of interaction-dominant systems are qualitatively or indeed quantitatively different from questions raised where an interaction-dominant system is not evidenced. We will address this in the final part of the paper.

Prior to a more detailed consideration of pink noise and interaction-dominant hybrid human-machine systems, it is important to emphasise the fundamental correlation between noise and cognition, and more specifically between noise and the distortion of cognition. We have to understand noise not only as an object of perception and cognition, but as partaking in the process of perception and cognition. Inquiry into distortion of cognitive processes is a necessary part of inquiring into the conditions of possibility of perception and cognition. Any philosophical inquiry into human agency must deal also with the state of indecision and confusion associated with noise. Any epistemological enquiry into the nature of knowledge, finally, must contend with the role of noise as lived ambiguity, indecision and error in communication processes – cognition is one such communication process as it entails the expression and exchange of information. Noise as a central component of the dynamics of all information systems [27] will have a dramatic impact on the manner in which our cognitive processes are technologically mediated. Despite its role as a *de jure* precondition for cognition, an enabling constraint, noise is a term that still carries many negative connotations (unwanted signal, state of disorder or disturbance that does not contain meaningful data or information). These characterizations thus fail to recognise the multi-scale complexity of noise or its intrinsically functional relationship within cognitive, biological, social, political, and economic systems [28], as well as inferential reason (as a process of making generalisations based on data while taking into account uncertainty). From our perspective, it is evident that the emergence and increasing pervasiveness of human-machine cognitive hybrids augments the necessity of an analysis of noise in cognitive systems as cognitive processes are increasingly coupled with and are extended into the ever-expanding array of digital technologies and networks (e.g. [29]).

We now turn to the role of “pink noise” as constituting and indicating interaction-dominant systems before discussing some potential ethical considerations arising out of the formation of human-digital artefact couplings that can be characterised as interaction-dominant systems and subsequently, the consequences of our analysis for RRI approaches to enhancement via cognitive artefacts.

## Pink Noise and Interaction-Dependent Systems

### Extended Cognition and Sensory Substitution Devices

It is helpful here to first review the idea of “extended cognition” on which our claims about noise centre. Coarsely put, it is the idea that cognitive systems are extended beyond the boundary of a discrete organism. Cognition is not confined to the limits of our brain, but is attached to embodied sensorimotor processes which are a restraint upon cognition, and not *the* final limit of cognition. Cognition, subsequently, should be understood as a phenomenon that encompasses processes implicating brain, body, and environment. Moreover, the role of the brain in the process of cognition is not just as a sensory-machine that reacts specifically to certain stimuli or sensory modalities but rather a complex “task-machine,” that can, to a degree, re-establish function with input from other senses [30, 31]. This latter aspect is the domain of sensory substitution (SS), where touch or audition, for example, transmit information that is otherwise not available, due, for example, to a visual impairment. Sensory substitution devices (SSDs) have been available for a long time. A blind person’s cane, for example, translates environmental structure into haptic and proprioceptive feedback and sign language translates visual stimuli into language. There are numerous experiments testing if SSDs can become part of extended cognitive systems (e.g. [32–34]). These experiments have shown that sensory-substitution devices can indeed become part of extended cognitive systems and, additionally, these artefacts partially constitute the extended cognitive system. To prove this, researchers have looked at the changes in the information flow (between the nervous system and the devices) produced while the participants engaged with their environment during the task [35, 36]. These experiments used detrended fluctuation analysis (a method to measure structural information by quantifying the self-similarity of a time series in a system) and produced a signal that can be considered pink noise.

### Pink Noise

Pink noise is a type of variability in a data series that is neither random nor predictable, it has a fractal fluctuating structure. We can collect this data series, for example, by an experiment in which the participants have to press a key in response to a signal on a computer screen [37]. It is possible to measure the time it takes the participant to press the key as a result of noticing the signal (key-press response time), and the time it takes the participant to release the key to return to the waiting stance for the next trial (key-release response time). The two data series (key press and key release) are subjected to spectral analyses that can identify the pink noise in each separate data series. Pink noise is manifest in the inherent residual variability that remains after the average time interval that each participant produces (for each target interval) is removed from each trial series. Consequently, we can say that pink noise is revealed in the structure of the “background noise” of cognitive performance – the inherent variability of a participant’s cognition of passing time.

In the case of a cognitive system, the presence of pink noise indicates that the connections between the different parts are highly non-linear; that means small input changes result in counter-intuitively large changes in the output [35, 36, 38–40]. Time series of human performances (e.g. reaction time, memory retrieval etc.) expose patterns of variation with a structure based on self-similarity – this means that the shape looks like itself however much you zoom in or out, like Romanesco broccoli. This self-similar property is true for a type of patterns known as fractals. Fractals are termed infinitely complex because the more closely you look at the object, the more complex it appears. In normal geometry, shapes are defined by a set of rules (i.e. triangle: three straight lines that are connected). Fractal geometry also defines shapes by rules, nonetheless these rules are different. In fractal geometry a shape is made in two steps: first by making a rule about how to change a certain shape. This rule is then applied to the shape again and again, ad infinitum. In maths when you change something it is usually called a *function.* Thus, a *function is applied to a shape recursively*.

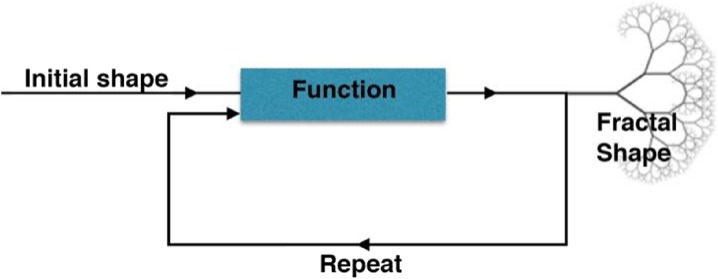



In its fractal fluctuating structure, pink noise expresses the iteration of convergent solutions – tending toward the same result – for common functional problems. Like the branching structure of a tree: from the bottom to the top of a tree, branches become thinner in diameter as they become more numerous. That is, a small piece of the tree looks to a certain extent like an entire tree. A large tree is a complex object, but it is formed by repeating a simple process over and over again.

Thanks to its pattern, pink noise illustrates optimal coordination among the components of a cognitive system and the task environment. Pink noise manifests both stability and adaptability, both attributes characteristic for healthy complex systems [41]. Numerous dynamical diseases (diseases that occur due to an abrupt change in the natural rhythms of the body, i.e. cardiac arrhythmia or epilepsy) have as a common form a transition away from healthy fractal variability and toward a loss of complexity in the dynamical unfolding of a system’s behaviour across time [42]. These fractal patterns pop up repeatedly in the natural world: physical, biological and economic systems exhibit pink noise [43, 44]. Because of this, some researchers describe it as being ubiquitous [41] and appearing when the components of a system are so firmly integrated with one another that their functions cannot be explained independently. Van Orden et al. [35] argue that if we can observe the activity of pink noise during the human performance of a list of cognitive tasks, this demonstrates that the cognitive system is fully embodied and includes aspects that are extended to the periphery of the organism.

Fractal dynamics inform us about the coordination of component processes in living systems. In this respect, it is very revealing that the common fractal signature of a healthy functioning system is found extensively in natural systems that self-organise their behaviour. Self-organisation requires a specific type of interaction to coordinate the processes that must perform together. The correct form of this interaction equipoises competitive and cooperative processes to produce an adaptive and flexible functional configuration or critical state, hence the scientific term “extended criticality.” The interaction that leads to critical states has been calculated for simple physical systems but it is also valid for a working hypothesis for more complex biological and cognitive behaviour. The name for this kind of interaction among component processes is interaction-dominant dynamics. The key fact of interaction-dominant dynamics is that system components change each other’s dynamics to coordinate their collective behaviour [45] to the extent that delineation between functional components is not rigid but rather characterised by its plasticity.

Following from the previous two sub-sections it is clear that coupling between human perceiver and artefact in the case of some sensory-substitution devices can be constitutive of the pink noise (in the analysis of information flow in the coupling relation) that is indicative of interaction-dominant systems. The next sub-section will show that this relation applies to a wider class of human-cognitive artefact relations, including digital artefacts and human-machine interfaces.

### Computer Mouse Experiments

Dotov et al. [33] have shown that cognitive systems can be made to extend beyond the outer limits of the organism to include digital artefacts in a manner that can be characterised as interaction-dominant. In their experiments, the participants played a simple video game that involved controlling an object on a computer screen using a computer mouse. At random moments during one-minute trials, the connection between the mouse and the object it controls was interrupted momentarily before returning to normal. While the mouse was operating normally, they found evidence of pink noise at the computer mouse interface (hand motions followed the mathematical pattern of pink noise), which diminished during the interruption. Using motion-tracking equipment they recorded the three-dimensional trajectory of the hand-tool system (human and interface); the hand motions of the computer mouse exhibited the nested fractal structure of pink noise (formal and statistical self-similarity). This is where the noise is manifest: at the interface of body and tool [33], because “Pink [1/f] noise cannot be encapsulated; it is not the product of a particular component of the mind or body.” ([35], p. 345). Dotov et al. [33] applied an analysis that has been used to establish long-range correlations in the time series which are expressed as pink noise: these long-range correlations stand for long-term dependencies in a signal between the present observation and a large set of previous observations. Thus, the presence of long-range correlations implies the presence of multiple, nested timescales in the system, responsible for the emergence of patterns in the system. Additionally, Dotov et al. [46] also prepared the experiment to measure physical indicators of stress, such as respiration rate, heart rate, and galvanic skin response (changes in the electrical activity of the skin triggered by emotional or physiological responses). They found an increase in all three at precisely the same moment when the mathematical pattern transitioned from pink noise to chaos.

This shows that, under optimal connection, the computer mouse (a digital cognitive artefact comprising a human-machine interface) is part of the smooth functioning interaction-dominant system involved in the task and that during the mouse disruption the pink noise at the computer mouse interface decreases momentarily, pointing out that the mouse is no longer part of the extended interaction-dominant system. Interaction-dominant dynamics [47] express the plasticity of the system’s elements and of the communication modes among these elements. Coordinated processes (like the use of a computer mouse to point at something) can alter the integrative action of components to the extent that it is hard, and sometimes unfeasible, to assign tightly defined and unique roles to specific elements. An interaction-dominant system entails that any singular component of the system interacts through the system as a whole, remodelling the dynamics of the other components and overriding the dynamics that the components would exhibit separately. In interaction-dominant systems we cannot treat the components of the system in isolation. Because of the extensive feedback in interaction-dominant systems, one cannot isolate any one component to determine with exactitude what function it has in relation to a particular behaviour. Since interactions dominate, organisation is emergent and depends on the context. The parts that constitute the system arrange themselves according to the current demands of context and perform functions according to this. That is: components can flexibly tie together or split to befit the changing conditions for a given task [48]. Thus, organisation in an interaction-dominant system is an emergent coordination, and instead of responding to local divisions and parts, this coordination emerges in accordance with ongoing changes in information flow [49]. Because of these ongoing changes, the behaviour of the components in any particular interaction-dominant system is not predictable from their behaviour in isolation or from their behaviour in some other interaction-dominant system. In other words, interaction-dominant systems are not modular in their design nor in terms of “modular cognitive architecture”; they are in a deep way unified in that the accountability for the system behaviour is scattered across all of the components.

### The Ready-to-Hand Computer Mouse

As a result of changing the focus of attention from the information flow to the presence of pink noise, van Orden et al. [35] argued that the participant-computer system formed an interaction-dominant system, and also provided some empirical confirmation for an aspect of Heidegger’s transition from present-at-hand to ready-to-hand modes of experience [50]. Present-at-hand refers to our theoretical understanding of a world constituted of objects as standing apart from or against the subject or agent; it is a mode of comprehension. But with the “ready-to-hand” notion of experience, Heidegger argued that people do not notice familiar, functional tools, but instead they “see through” them to a task at hand, for precisely the same reasons that one does not think of the way that one’s fingers hold the pen while writing. *The tools are us*. Similarly, proponents of extended cognition have argued that the artefacts into which cognition is extended must be functionally transparent to the agent [11], they must be used without the agent actively thinking about what they are doing, i.e. how they are using the artefact or incorporating it into their cognitive or motile processes. The distinction that this builds is between an artefact rendered transparent and integrated into cognitive or motile processes and an object that is conspicuous and “stands against” the controlling agent. “Standing against” does not necessarily imply a hostile relationship, it is a general term used in the phenomenological literature for the epistemological-experienced status of objects in the world vis-à-vis the subject, but it does imply an experienced distance between subject or agent and object.

The French philosopher Merleau-Ponty [51] built upon this analysis to argue that bodily prosthetics, like a walking cane, are, with use, integrated into what he called the “body schema”, an integrated system of bodily motile possibilities functioning both at the level of unconscious sensorimotor processes and consciously experienced movement. From the perspective of both passive (ones we are not aware of) and active (ones we are) conscious processes, the prosthesis becomes part of the body schema. This form of interaction-dominant coupling could be interrupted if for example the stick was accidentally dropped or struck from the hand, returning it to its phenomenological status of conspicuous external object. The discussion of digital artefacts that we engage in here proceeds on much the same grounds. What differs are the descriptive mechanisms for demonstrating the interaction-dominant character of the coupling.

Returning to the experimental setting, when the computer mouse was controlling correctly the on-monitor pointer, the participants experienced their control of the object in the video game, they could see through the tool to focus on the task they were performing, *the presence of pink noise was evident and the computer mouse was experienced as ready-to-hand*. When the connection between mouse movements and the on-screen control of the object was perturbed, the participants were concerned about the performance of the mouse, they were no longer able to see through the malfunctioning tool, there is no presence of pink noise and they experienced it as present-to-hand. The phenomenological accounts of ready-to-handedness or prosthetic integration into the body schema are equally applicable here in the case of a digital artefact and accompany the presence and absence of pink noise in the analysis of information flow.

The phenomenological accounts developed by Heidegger [50] and Merleau-Ponty [51] are also significant because they bring to the fore several important characteristics of interaction-dominant systems. The accounts of “ready-to-hand” tools and prostheses integrated in body schemata do assume a central subjective controller, an agent whose intentional relations with the surrounding world drive the processes in question, even if biases can be built into artefacts that condition their usage and potential integration into a body schema. Philosophers of technology have pointed out that artefacts are not value-neutral but have affordances and indeed values built into their design. These embedded histories may or may not be apparent to the designers themselves, but often – one can imagine examples where this is not the case, such as when an artefact is transferred outside of its originally intended use context, in play for example – condition the use of the artefact, undermining any claims to absolute sovereignty on the part of a central controlling-acting agent. Nonetheless, the phenomenological example brings to the fore the question of agency in interaction-dominant systems. Closely linked to the question of agency is the issue of passivity in the emergence of interaction-dominance. In both the example of the ready-to-hand tool and the prosthesis that has been incorporated into the body schema, the integration into cognitive and motile processes happens in a manner that is termed passive, or not fully present to consciousness. In other words, the agent or subject is not consciously aware (in the way that we normally use the term) of the full integration of the artefact into its cognitive and motile processes. In fact, as many phenomenologists like to point out, when conscious attention is turned to the relation between the body and the artefact the integration is broken and the artefact appears suddenly conspicuous and often unwieldy. What we wish to argue is that in some settings this conspicuousness, which can also be characterised as or via the concept of noise, qua disruption of information flow, can be an epistemic and ethical virtue.

### Pink Noise as the Evidence of an Extended Cognitive System

The presence of pink noise during the performance of human-artefact coupling as demonstrative and constitutive of an extended and interaction-dominant system cognitive system, where the device is not merely causally related to the system but is constitutive of the system as such illustrates the relevance of noise, and specifically the sub-type pink noise, to an understanding of cognitive processes. This, along with the significant amount of methodology from complex systems theory that is being brought into cognitive sciences, provides good reason to doubt some of the methodological truisms that cognitive sciences students are commonly taught, namely that a good experimental design in the cognitive sciences looks for the minimization of error variance (noise). A significant amount of nonlinear dynamical modelling techniques refute this conception by taking the structure of noise to be the primary data, as we do here. We understand data as the outcomes of observations, measurements, and procedures that the scientists carry out. Quite often these outcomes will be measures of fluctuations (as signals depending on the theoretical framework), which is fundamentally noise.

In cases like the experiments by van Orden et al. [35] and Anderson et al. [47], noise no longer should be received as meaningless fluctuations that can be overlooked or ignored as second order data, nor confused as uncertainty. Precisely because here, *pink noise is the evidence* (at the core of the system) that humans and computers together can comprise unified interaction-dominant systems: *pink noise designates a unified system of parts.* It is important to emphasise the temporal dimension of interaction-dominance alongside the functional indiscernibility of causal components within an interaction-dominant system. Chemero [52, 53] very often gives the example of walking. When we walk on a level path, our stride length will appear to be mostly the same, but there are subtle variations. These variations create a system that has a “long memory”. It is the very same long memory processes with long-term correlations that exhibit pink noise fluctuations. The way we walked twenty paces ago affects the pace we are about to take. If the system were not interconnected in the way that it is, it would show randomness. Pink noise is neither regular nor random; it is an irregular, fractal pattern that resembles itself on large and small scales and stands for a system whose parts interact densely in real time.^7^

## The Many Virtues of Noise: Heuristic, Epistemic and Ethical

Let us return briefly to the key aspect of the summary definition of interaction-dominant systems: This entails that any singular component of the system interacts through the system as a whole, remodelling the dynamics of the other components and overriding the dynamics that the components would exhibit separately. The result is a functional and temporal indiscernibility and plastisity of causal components in an interaction-dominant system. The experiments discussed in the previous section demonstrate that the emergence of such systems is possible under quite routine conditions. Our contention here is that interaction-dominant systems, as emergent but common occurrences, can, by the fact that they do not consist of discernible causal, temporal or functional components, complicate or render impossible the assignment of agency or potentially responsibility as well as our understanding of autonomy in ever more prevalent human-digital artefact couplings. This is in part illustrated by several counter examples. Recall the previous example of a walking stick used as a sensory substitution device being integrated in the body schema of an agent to an extent that when in use an interaction-dominant system emerges. The light-weight stick may offer affordances [52] (we understand affordance here in a very basic sense as a perceptually manifest possibility of an object for action in an environment) for more nefarious use, for example thwacking others on the street. In such cases, despite the existence of an interaction-dominant system having emerged, the assignment of agency and responsibility for the action is not in doubt. Though the intensity of the extension is such that functional delimiting of parts is not possible, there remains little doubt of a central, subjective in this case, controller who is the agent of the action and hence the potential subject of responsibility. When the coupled artefact makes use of algorithms for problem solving, the situation *may* be different. It is helpful here to parse the discussion through Floridi’s and Sanders’ [18] discussion of distributed morality.

Drawing on theories of distributed cognition wherein a set of cognitive agents has knowledge that no one individual within the set has, Floridi builds an account where a set of morally neutral or negligible acts interact to create a morally salient act as an emergent property of the interaction – when two potentially neutral states or acts interact in the right way the result of the interaction is morally salient. It is important here to understand the salient act in two possible ways: on the one hand, it might be an emergent property of the interaction or it could be the cumulative effect of otherwise below moral salience threshold acts accumulating to pass the threshold of salience. The analysis here is decidedly consequentialist, or “receiver-perspective” since salience is gauged in terms of overall impact on the environment and its inhabitants, not on actor intentions. Such instances of distributed morality can go both ways, i.e. toward positive and negative evaluation. Floridi provides the example of consumer driven corporate responsibility programmes which require a critical mass of participation to become salient.

What distinguishes cases of distributed morality from interaction-dominance? It is certainly possible that distributed cognition or morality networks can be interaction-dominant, but they are not necessarily so. In the examples discussed by Floridi and Sanders [18], the delimiting of discrete causal components and behaviours is possible, responsibility for certain temporal or functional events in the process of a network interaction or process can still be assigned. This is not the case in an interaction-dominant system. We should be careful to remain specific in our understanding of what an interaction-dominant system is and how it is empirically identified, hence the importance of pink noise in this discussion. Heersmink [5, 11] helpfully contrasts Floridi’s distributed morality approach with the more actor-network theory influenced notion of “distributed agency” developed by Verbeek [57]. Verbeek’s account confronts what he argues is the non-value-neutrality of certain artefacts in context. It is not so much that values or designers’ intentions are embedded in artefacts in a way that directly impacts a morally relevant context, but rather that aspects of technology become value-charged within certain contexts to the extent that it is not possible to say that the artefact is value-neutral as the meaning of its functionality can only appear in a context. Heersmink, following Verbeek, refers to the example of an ultrasound machine noting that the enlarged size of the imaging, the possibility of discerning gender, in other words, the personification of the foetus via the imagining technology, is not value-neutral and that this can only be assessed in context. Examples such as this may be morally salient, but may still lack the same characteristic of interaction-dominance, namely the specific form of functional integration demonstrated by the appearance of pink noise. The lack of interaction-dominance is significant because the possibility for a clear if not totally transparent delineation of functional causality and competence within a system or network allows for a clearer, if not necessarily transparent, assessment of responsibility.

We can imagine examples where the demonstration of interaction-dependence, particularly in the relation between human and digital cognitive artefacts (artefacts making use of decision making and problem solving rules) has particular significance in assessing responsibility. There are reports of systemic racial bias in some decision-making algorithms, for example the COMPAS system used to assess the likelihood of recidivism for accused criminals. Speilkamp [58] summarised the findings of ProPublica [59]:

ProPublica, a Pulitzer Prize winning not-for-profit news organisation, analysed risk assessment software known as COMPAS. It is being used to forecast which criminals are most likely to reoffend. Guided by such forecasts, judges in courtrooms throughout the United States make decisions about the future of defendants and convicts, determining everything from bail amounts to sentences. When ProPublica compared COMPAS’s risk assessments for more than 10,000 people arrested in one Florida county with how often those people actually went on to reoffend, it discovered that the algorithm “correctly predicted recidivism for black and white defendants at roughly the same rate.” But when the algorithm was wrong, it was wrong in different ways for blacks and whites. Specifically, “blacks are almost twice as likely as whites to be labelled a higher risk but not actually re-offend.”

Algorithmic decision making can be biased for a number of reason, including that often unconscious or implicit biases of those writing the algorithms are built into their rule making structures, or for reasons unknown to engineers because the mechanisms of the algorithm have been blackboxed. In cases such as COMPAS, the digital artefact is supposed to provide guidance to a human decision maker who, in these cases at least, remains the central controlling agent (to use the language of extended cognition). However, if usage of the interface is such that there is evidence of the emergence of an interaction-dominant system the temporal and functional delineation of competencies within the decision making process may be difficult to discern (we introduce this as a hypothetical, not as an actual assessment of the COMPAS system). In situations where such delineations are essential for the possibility of assigning legal or moral responsibility and also for the possibility of appeal due to evidence of bias somewhere in the components of the system, prior to the formation of the interaction-dominant systems (or after the fact) the appearance of pink noise is not only a potentially useful heuristic, but a possible canary in the proverbial mine. We do not mean to suggest that testing for pink noise is a way of overcoming the issues pertaining to the use of automated decision-making systems in the criminal justice system, nor even that bias introduced by the algorithms used by systems such as COMPAS may somehow be worse than unextended (into digital cognitive artefacts) human biases. We could also envision a situation wherein biases embedded in computer algorithms could be corrected for by other computer algorithms. Rather, that pink noise is a potentially helpful and important indicator of interaction-dominant relations, and that the latter may be undesirable in contexts where the identification of functional and temporal causal accountability is considered required. Pink noise is a heuristic key to the phenomenon that we called, at the beginning of this article, the over-extended mind. Hence, we think that the role of pink noise is potentially important further upstream in the design process and has lessons for the responsible or value-sensitive design and innovation of digital cognitive artefacts for the purposes of cognitive or other forms of enhancement. The over-extended mind and with it the role of pink noise point to the importance of distance and functional demarcation as an epistemic, ethical, and even social-political virtue in the design of interfaces between humans and digital cognitive artefacts. In other words, it points to *noise qua disturbance* in human-digital artefact coupling as a potential epistemic, ethical and social-political virtue in value-sensitive design. While interaction-dominance can certainly be a virtue in the case of sensory substitution prosthetics (e.g. the walking stick) it is less likely to be so when the coupled artefact has its own decision-making processes and rules that may not be transparent or available to other nodes in the network and when the delineation of functional competence and linear temporal relations is central to the moral, political, or legal evaluation of an action or behaviour. The goal of seamless integration with digital artefacts may have unforeseen negative consequences, while distance, disruption of information flow, and distraction, classical noisy enemies of cognition, may turn out to be virtues after all as the extension of morally, legally, and politically salient decision making and behaviours into digital artefacts becomes increasingly pervasive. Thought and reflection, as opposed to cognition, are after all often noisy and make use of resistances and interference to become more adaptive. As we look to digital artefacts to enhance all sorts of capacities, this desirability of distance, demarcation, and even disruption may be worth remembering.

In his seminal paper, “Do artifacts have politics?”, Langdon Winner [60] argued convincingly that they do. A further difficulty emerges when one faces artefacts or technical assemblages that make it increasingly difficult to unravel the politics embedded in them from one’s own. This is a situation that we think is made more likely and more prevalent by the increasing pervasiveness of digital cognitive artefacts and, in some cases, the emergence of ethically salient interaction-dominant systems. In this context, some noise between us and our digital tools may not just help to discern both responsibility as well as, in the case of pink noise, potential issues in assessing certain type of responsibility, but may also be a key indicator in the responsible innovation of human-machine interfaces.

## Footnotes

1 We understand digital cognitive artefacts simply as cognitive artefacts (defined below) that are digital in nature or incorporate digital processes.

2 Cognitive computing describes technology platforms that combine machine learning, reasoning, natural language processing, speech, vision, human computer interaction, that mimic the functioning of the human brain and help to improve human decision making (http://www.predictiveanalyticstoday.com/what-is-cognitive-computing/).

3 See, https://www.partnershiponai.org/

4 “Responsible Innovation is a process that seeks to promote creativity and opportunities for science and innovation that are socially desirable and undertaken in the public interest. Responsible Innovation acknowledges, that innovation can raise questions and dilemmas, is often ambiguous in terms of purposes and motivations and unpredictable in terms of impacts, beneficial or otherwise. Responsible Innovation creates spaces and processes to explore these aspects of innovation in an open, inclusive and timely way. This is a collective responsibility, where funders, researchers, stakeholders and the public all have an important role to play. It includes, but goes beyond, considerations of risk and regulation, important though these are.” https://epsrc.ukri.org/research/framework/ (last accessed 14 September 2018)

5 “NWO-MVI maps and facilitates the incorporation of ethical and societal aspects of technological innovations early on in the design process. Our aim? Responsible innovations that enjoy broad support in society.” https://www.nwo-mvi.nl/ (last accessed 14 September 2018)

6 Algorithms can be understood technically as sets of “encoded procedures” that “transform input data into a desired output based on specified calculations” ([15], p. 167). In more general language, an algorithm is the result of ‘what is to be done’ with ‘how it should be done’, or rather, ‘defining a problem’ and ‘naming the steps necessary to solve that problem’ ([16], p. 17). Algorithms, insofar as they assign relevance to sets of data, have a fundamental normative function. We are grateful to Suzanna Kraak [17] for making this point.

7 Thornton and Gilden [54] and Torre and Wagenmakers [55] argued that 1/f-like scaling might result from a component-dominant system – components which interact in sequence as in a machine, rather than an interaction-dominant system. Recently, however, Ihlen and Vereijken [56] have shown that the presence of multifractality demonstrates definitively that a system is interaction-dominant. Ihlen and Vereijken reanalysed the data from van Orden et al. [35], and showed that it is multifractal.
